# Dual roles of WISP2 in the progression of hepatocellular carcinoma: implications of the fibroblast infiltration into the tumor microenvironment

**DOI:** 10.18632/aging.203424

**Published:** 2021-09-08

**Authors:** Qingan Jia, Yaoyao Zhang, Binghui Xu, Xia Liao, Yang Bu, Zihan Xu, Xianglong Duan, Qiangbo Zhang

**Affiliations:** 1 Institute of Medical Research, Northwestern Polytechnical University, Xi’an 710072, China; 2 Department of Nutrition, The First Affiliated Hospital of Xi’an Jiaotong University, Xi’an 710061, China; 3 Department of Hepatobiliary Surgery, General Hospital, Ningxia Medical University, Yinchuan 750001, China; 4 Department of Burns and Plastic Surgery, Affiliated Shaanxi Provincial People’s Hospital, Northwestern Polytechnical University, Xi’an 710068, China; 5 Second Department of General Surgery, Shaanxi Provincial People's Hospital Affiliated Hospital of Northwestern Polytechnical University, Xi’an 710068, China; 6 Cheeloo College of Medicine, Shandong University, Jinan 250012, China; 7 Department of General Surgery, Qilu Hospital, Shandong University, Jinan 250012, China

**Keywords:** HCC, WISP2, HMGB1, fibroblast, prognosis

## Abstract

The dismal outcome of hepatocellular carcinoma (HCC) patients is attributable to high frequency of metastasis and. Identification of effective biomarkers is a key strategy to inform prognosis and improve survival. Previous studies reported inconsistent roles of WISP2 in carcinogenesis, while the role of WISP2 in HCC progression also remains unclear. In this study, we confirmed that WISP2 was downregulated in HCC tissues, and WISP2 was acting as a protective factor, especially in patients without alcohol intake using multiple online datasets. In addition, we reported that upregulation of WISP2 in HCC was related to inhibition of the malignant phenotype *in vitro*, but these alterations were not observed *in vivo*. WISP2 also negatively correlated with tumour purity, and increased infiltration of fibroblasts promoted malignant progression in HCC tissues. The enhanced infiltration ability of fibroblasts was related to upregulated HMGB1 after overexpression of WISP2 in HCC. The findings shed light on the anticancer role of WISP2, and HMGB1 is one of the key factors involved in the inhibition of the efficiency of WISP2 through reducing the tumour purity with fibroblast infiltration.

## INTRODUCTION

Hepatocellular carcinoma (HCC) has high incidence rates in China, which accounts for more than 50% of the total number of liver cancer cases and deaths in the world [1]. Even though many advanced strategies, including liver transplantation, molecular targeted therapies and immune-based treatments, the general prognosis of patients with HCC is still unsatisfactory [2]. Therefore, continued identification of new molecules for the development of combining targeted therapy is still urgently needed.

Cellular communication network (CCN) family are scaffolding proteins that may govern and balance the interconnection among individual signaling pathways. CCN proteins are a six-member family of cysteine-rich regulatory proteins that exist only in vertebrates, including CCN1 (cysteine-rich 61, CYR61), CCN2 (connective tissue growth factor, CTGF), CCN3 (nephroblastoma overexpressed, NOV), CCN4 (Wnt1-inducible signaling pathway proteins, WISP-1), CCN5 (WISP-2), and CCN6 (WISP-3) [3]. The diverse effects of physiological and pathological events are attributed to each structural domain of CCNs (with CCN5 lacking the CT domain): IGFBP, VWC, TSP-1, and CT [4]. Although CCN family members have highly consistent biological structures, these factors have differential expression and play specific roles in biology in different human cancers [5]. WISP2 differs from the other CCN family members due to a lack of the C-terminal domain, which has been shown to interact with extracellular cytokines, and receptors such as, integrins, EGFR, Notch 1 and LRP6 [6]. WISP2 can also interfere with the other CCN isoforms, influencing the function of these protein family members [7]. The expression and function of WISP2 are diverse in different human cancers. In breast cancer, WISP2 was reported as an oncogenic role [8]. By contrast, WISP2 is downregulated in human leiomyoma [9], pancreatic adenocarcinoma [10], salivary gland cancer [10], colorectal tumors [11], and gallbladder cancer [12], suggesting that it acts as a tumor suppressor. Up to now, the role of WISP2 in tumor progression also remains unclear in HCC [13].

In the present study, we analysed expression of WISP2 and the prognostic correlation in HCC patients using Oncomine, Kaplan–Meier plotter, and Gene expression profiling interactive analysis 2 (GEPIA2). We explored the role of WISP2 in HCC using the Cancer Cell Line Encyclopedia (CCLE) and gene microarrays and then assessed the correlation between WISP2 and stromal cells in tumour tissues using the Tumor IMmune Estimation Resource (TIMER). Finally, the role of WISP2 and its relationship with tumour purity and fibroblast infiltration were examined both *in vitro* and *in vivo*. Our studies revealed the anticancer role for WISP2 was conditional in HCC, and the efficiency was influenced by fibroblast infiltration in the tumor microenvironment (TME).

## MATERIALS AND METHODS

### mRNA expression, gene correlation, tumour purity, and immune infiltrate analysis in TIMER

TIMER is a comprehensive resource for analysis of immune infiltrates across diverse cancer types (https://cistrome.shinyapps.io/timer/) [14]. The expression levels of *WISP2* between tumour and adjacent normal tissues in different types of cancer were identified across all TCGA tumours via ‘Diff Exp’ module. The correlation between *WISP2* expression and immune infiltration, including B cells, CD4^+^ T cells, CD8^+^ T cells, neutrophils, macrophages, and dendritic cells, as well as a pairwise gene (CD34, PECAM, VCAM1, NT5E, ESM1, S100A4, VIM, and ACTA2) correlation in liver cancer were explored via ‘Gene’ and ‘Correlation’ modules, respectively.

### mRNA expression and survival analysis in GEPIA2

GEPIA2 is resource for analyzing the RNA sequencing expression data of 9,736 tumours and 8,587 normal samples from the TCGA and the GTEx projects [15]. WISP2 tumour/normal differential expression and the correlation between WISP2 expression and survival in diverse cancer types are analysed in GEPIA2.

### mRNA expression in cancer cell lines in the CCLE

WISP2 expression levels in cancer cell lines from diverse cancer types were examined using the CCLE (http://www.broadinstitute.org/ccle), which provides public access to genomic data, analysis, and visualization for more than 1,100 cell lines [16].

### Survival analysis in Kaplan–Meier plotter

The correlation between WISP2 expression and survival in liver cancer was analysed using the Kaplan–Meier plotter (http://kmplot.com/analysis/) [17]. The Kaplan–Meier plotter can assess the effects of 54,000 genes on survival in 21 cancer types. Gene expression data and RFS and OS information were downloaded from GEO, EGA, and TCGA.

### Protein-protein interaction (PPI) analysis in GeneMANIA

GeneMANIA is an online tool that predicts the function of genes and gene sets, including protein and genetic interactions, pathways, co-expression, co-localization, and protein domain similarity, in GeneMANIA [18] (https://genemania.org). WISP2 and CTGF were used as queries to predict PPIs, and the prediction output graphically shows a network that depicts the relationships between genes in the list.

### Clinical features analysis in LinkedOmics database

The relationship between *WISP2* expression and clinical features in liver cancer patients was analysed using the LinkedOmics database (http://www.linkedomics.org), which is a publicly available portal that includes multi-omics data from all 32 TCGA Cancer types [19]. Based on the platform, a statistical analysis of the correlation between *WISP2* expression and clinical features of HCC was performed.

### cDNA microarray analysis

cDNA microarrays were performed using the Human OneArray^®^ (Phalanx Biotech Group, San Diego, CA, USA) to evaluate the alterations of expression profiling. Total RNA was extracted from Hep3B-WISP2 and Hep3B-Vector cells and the isolations and microarray analyses were performed in triplicate according to the manufacturer’s instructions. All data was uploaded to the Gene Expression Omnibus (GSE134563).

Patients and tissue microarray analysis, cell lines culture, vector construction and transfection, various functional assays of WISP2 *in vivo* (animal models with subcutaneous xenografts) and *in vitro* (migration, invasion, and proliferation) were all exhibited in Supplementary Materials.

### Statistical analysis

Graphics were drawn using GraphPad Prism version 6 (GraphPad Software, La Jolla, CA, USA). Statistical analyses were performed using SPSS 15.0 for Windows (SPSS). A *p*-value <0.05 was considered statistically significant.

### Ethics approval

Animal protocols were approved by the Medical Experimental Animal Care Commission of Northwest Polytechnical University, and all methods were performed in accordance with the relevant guidelines.

## RESULTS

### WISP2 mRNA levels was lower in tumour tissues of HCC compared with normal tissues, but the antitumor role of WISP2 is conditional

**Figure 1:**
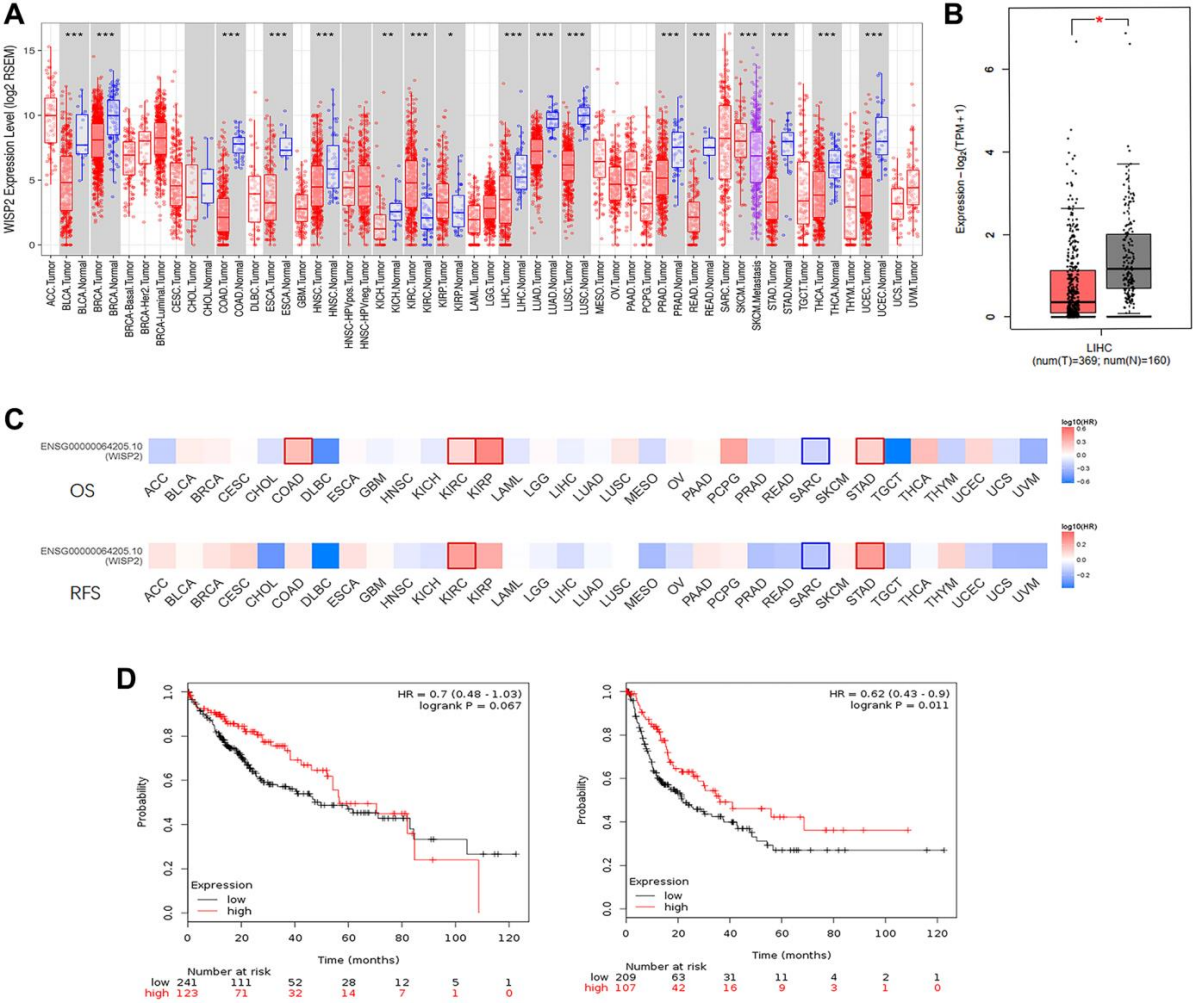
**WISP2 mRNA level is lower compared with normal tissues, and low expression of WISP2 is associated with poor prognosis.** (**A**) TIMER database was used to evaluate the WISP2 RNA-seq data in human cancers, and the expression of WISP2 was lower in most human cancers, including HCC. (**B**) The expression of WISP2 was lower in tumour tissues of HCC than normal tissues in GEPIA2 database. (**C**) The role of WISP2 on prognosis varied in 33 human cancers, and in HCC, WISP2 had a protective role from GEPIA2 database. (**D**) The protective role of WISP2 in HCC was confirmed using the Kaplan–Meier plotter, and low expression of WISP2 is associated with poor prognosis.

Using the TIMER and GEPIA2 database to evaluate the RNA-seq data, expression of WISP2 was found to be significantly downregulated in most human cancers, including HCC, compared with the associated normal tissues. Only in kidney renal clear cell carcinoma (KIRC) and kidney renal papillary cell carcinoma (KIRP) were WISP2 expression significantly higher than that in adjacent normal tissues (Figure 1A, 1B). To explore the role of WISP2 on prognosis, 33 human cancers were included using the GEPIA2 database. The role of WISP2 on prognosis varied in different types of cancers. In HCC, WISP2 had a protective role, as high expression was associated with better prognosis for this cancer type, although there was no significant statistical difference (Figure 1C).

To better understand the role of WISP2 in HCC prognosis, the Kaplan–Meier plotter was used. The HCC patients in the WISP2-high group had longer OS than the WISP2-low group, although there was no significant difference (HR = 0.7, *p* = 0.067). The patients in the WISP2-high group had a significantly longer RFS compared with those in the WISP2-low group (HR = 0.62, *p* = 0.011; Figure 1D). These results revealed WISP2 was acting as a protective factor and the level of WISP2 was decreased in HCC.

We evaluated the effects of clinicopathologic characteristics on the prognosis of HCC patients with different WISP2 expression levels. High expression of WISP2 was associated with longer OS in female patients (HR = 0.52, *p* = 0.026). Specifically, high expression of WISP2 mRNA was correlated with longer OS in stage 2 (HR = 0.37, *p* = 0.01) and longer RFS in stage 1 (HR = 0.5, *p* = 0.013) in HCC patients. High WISP2 expression was correlated with longer OS in grade 1 (HR = 0.3, *p* = 0.023) and 3 (HR = 0.45, *p* = 0.017) patients. In addition, high WISP2 expression was correlated with longer RFS in patients with no vascular invasion (HR = 0.57, *p* = 0.028), T1 (HR = 0.49, *p* = 0.0078), and T2 (HR = 0.36 *p* = 0.018) according to the criteria of the American Joint Committee on Cancer (AJCC). These results suggest that WISP2 expression levels can significantly influence the prognosis in most human cancers, but the role of WISP2 in HCC is conditional (Table 1).

**Table 1:** Correlation of WISP2 mRNA expression and clinical prognosis in HCC with different clinicopathological factors according to the Kaplan–Meier plotter.

**Clinicopathological characteristics**	**No. of patients**	**Overall survival (OS)** **(*n* = 364)**		**Relapse-free survival (RFS)** **(*n* = 313)**	
Sex		HR (95% CI)	*p*	HR (95% CI)	*p*
Male	250	0.66 (0.38–1.14)	0.14	**0.58 (0.37–0.91)**	**0.016**
Female	**121**	**0.52 (0.29–0.93)**	**0.026**	0.52 (0.25–1.09)	0.077
Stage					
1	171	0.68 (0.36–1.29)	0.23	**0.5 (0.29–0.87)**	**0.013**
2	86	**0.37 (0.17–0.82)**	**0.01**	0.21 (0.03–1.76)	0.12
3 + 4	90	0.058 (0.33–1.03)	0.059	0.48 (0.23–1.02)	0.051
Grade					
1	55	0.43 (0.16–1.17)	0.091	**0.3 (0.1–0.9)**	**0.023**
2	177	0.67 (0.4–1.15)	0.14	0.75 (0.46–1.23)	0.26
3	122	0.52 (0.24–1.13)	0.094	**0.45 (0.23–0.88)**	**0.017**
AJCC–T					
1	181	0.73 (0.4–1.33)	0.31	**0.49 (0.29–0.84)**	**0.0078**
2	94	**0.43 (0.21–0.9)**	**0.022**	**0.36 (0.15–0.86)**	**0.018**
3	80	0.59 (0.32–1.08)	0.083	1.67 (0.8–3.52)	0.17
Vascular invasion					
none	205	0.71 (0.39–1.28)	0.25	**0.57 (0.35–0.95)**	**0.028**
micro	93	1.6 (0.74–3.47)	0.23	0.78 (0.39–1.57)	0.48
Alcohol intake					
Yes	117	0.53 (0.25–1.12)	0.089	0.65 (0.33–1.28)	0.21
No	205	**0.61 (0.39–0.97)**	**0.033**	**0.56 (0.35–0.9)**	**0.016**
Hepatitis virus					
Yes	153	0.67 (0.34–1.34)	0.26	**0.56 (0.34–0.93)**	**0.023**
No	169	**0.51 (0.31–0.84)**	**0.0065**	0.54 (0.29–1.01	0.052

Bold text means the difference has statistical significance (*p* < 0.05).

### Upregulation of WISP2 in HCC is related to inhibited malignant phenotype *in vitro*

**Figure 2:**
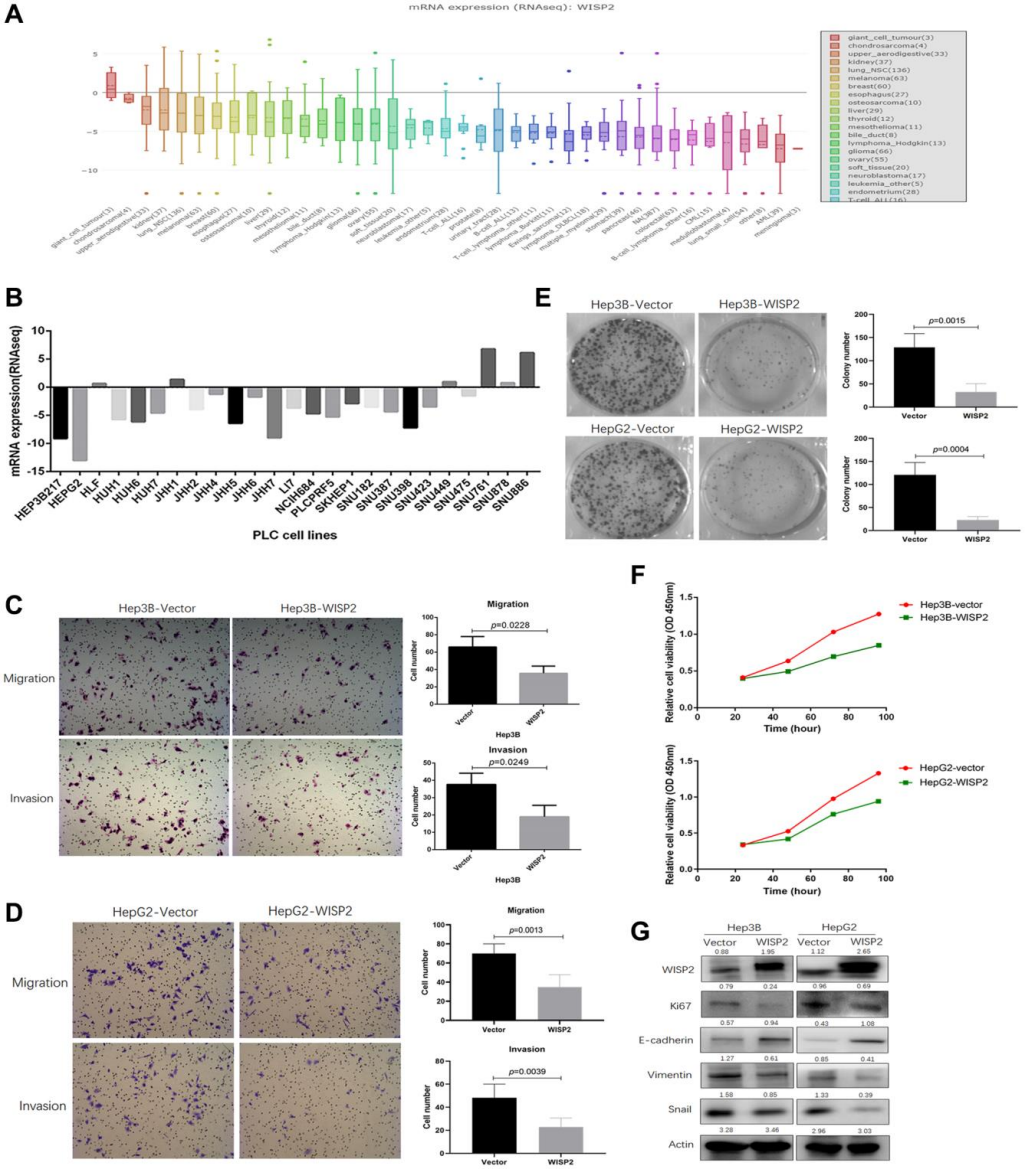
**Upregulation of WISP2 in HCC is related to inhibition of the malignant phenotype *in vitro*, while no difference in proliferation was found in subcutaneous tumorigenesis *in vivo*.** (**A**) The expression of WISP2 was diverse in different tumour cell lines in the CCLE database. (**B**) The expression of WISP2 in 76% HCC cell lines, including Hep3B and HepG2, were low in CCLE database. (**C**) The expression of WISP2 was determined in Hep3B and HepG2, and confirmed using immunoblotting and immunocytochemistry. (**D**) Overexpression of WISP2 significantly inhibited the migration and invasiveness in Hep3B and HepG2 HCC cells. (**E**) Proliferation in Hep3B and HepG2 HCC cells that overexpress WISP2 or control cells was examined using a plate colony formation assay, and overexpression of WISP2 significantly impaired the colony formation. (**F**) Proliferation was assessed using a CCK8 assay, and overexpression of WISP2 significantly inhibited the proliferation in Hep3B and HepG2 HCC cells. (**G**) The expression of Ki67, vimentin, and Snail were significantly downregulated, and the epithelial cell surface marker E-cadherin was upregulated in Hep3B and HepG2 cells that overexpressed WISP2.

To determine the precise function of WISP2, we first evaluated the expression of WISP2 in different human tumour cell lines from the CCLE, revealing the significant differences in the expression of diverse tumors (Figure 2A). We further analysed the WISP2 expression in 25 HCC cell lines from the CCLE, and found that the expression of WISP2 was low in 76% HCC cell lines, especially in Hep3B and HepG2 cells (Figure 2B).

In addition, we stably overexpressed WISP2 in Hep3B and HepG2 cells, and confirmed WISP2 expression using immunoblotting (Figure 2G). Hep3B-WISP2 and HepG2-WISP2 were compared with the associated control cells. Overexpression of WISP2 significantly inhibited the migration (35.67 ± 8.33 vs. 66.05 ± 12.51, *p* = 0.0228) and invasiveness (19.04 ± 6.56 vs. 37.67 ± 6.51, *p* = 0.0249) of Hep3B cell lines compared to the vector control (Figure 2C). Overexpression of WISP2 significantly inhibited the migration (34.80 ± 12.95 vs. 70.12 ± 10.02, *p* = 0.0013) and invasiveness (22.81 ± 7.86 vs. 48.25 ± 11.82, *p* = 0.0039) of HepG2 cell lines compared to the vector control (Figure 2D). We next examined the proliferative ability of these cells using a colony formation assay. Overexpression of WISP2 significantly impaired the colony formation of Hep3B cell lines (32.50 ± 18.05 vs. 128.81 ± 29.81, *p* = 0.0015) and HepG2 cell lines (13.13 ± 7.44 vs. 120.81 ± 26.86, *p* = 0.0004) compared to the vector control (Figure 2E). This proliferation inhibition following overexpression of WISP2 in Hep3B and HepG2 cell lines was confirmed with a CCK8 assay (Figure 2F).

We also evaluated markers related to the epithelial-mesenchymal transition (vimentin, Snail, and E-cadherin) as a measure of migration and invasiveness and Ki67 as a proliferation marker at the protein level in HCC cells. The expression of Ki67, vimentin, and Snail were significantly downregulated, and the epithelial cell surface marker E-cadherin was upregulated in Hep3B and HepG2 cells that overexpressed WISP2 (Figure 2G). All of these results suggest that WISP2 expression is acting as a protective factor *in vitro*.

### The increased infiltration of fibroblasts plays a negative feedback role in inhibition of the efficiency of WISP2 *in vivo*

**Figure 3:**
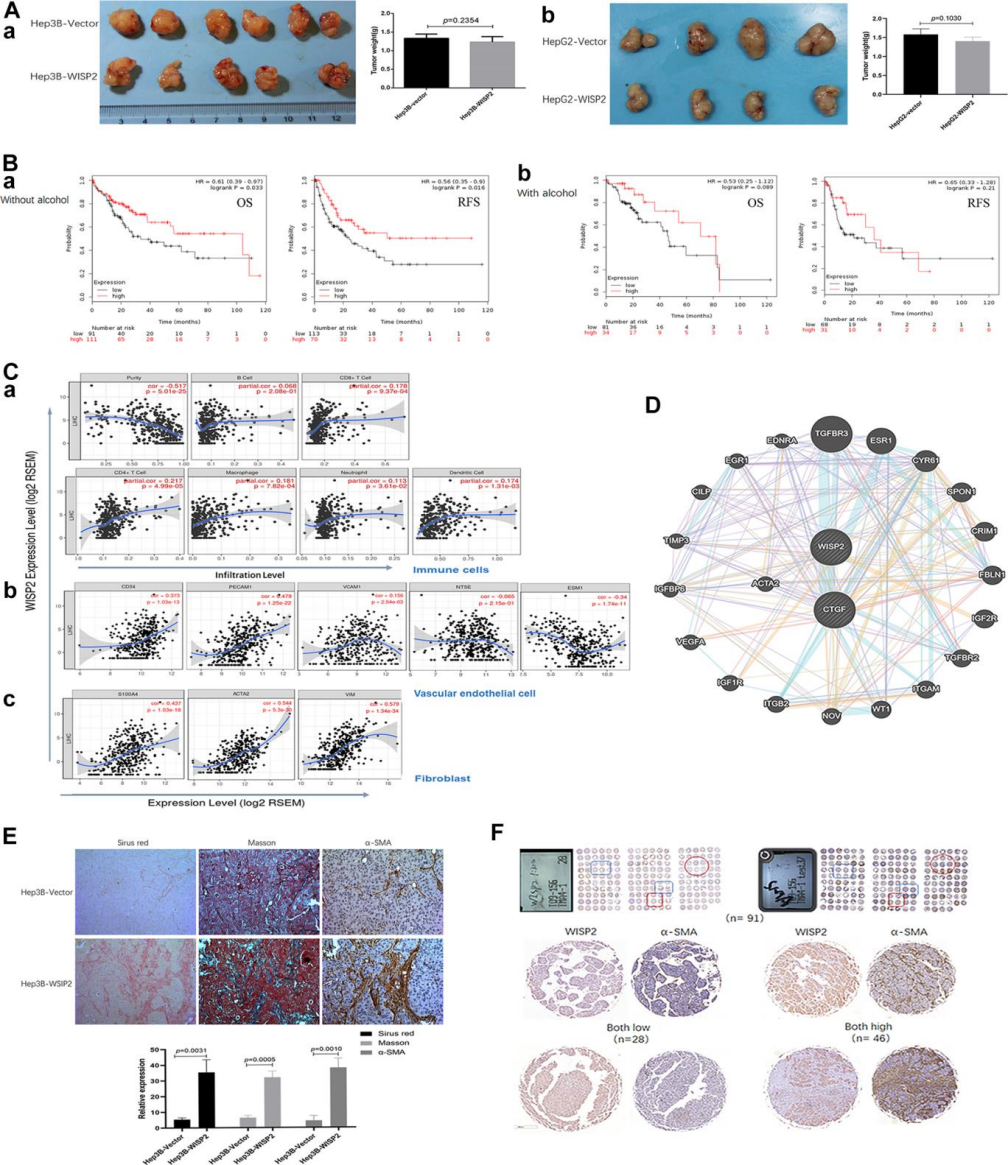
**Upregulation of WISP2 is related to tumour purity, and the infiltration of fibroblasts into HCC tissues exerts a negative role in HCC progression.** (**A**, **a**) In nude mouse models, subcutaneous tumour weights did not differ in mice injected with Hep3B-WISP2 and Hep3B-Vector. (**A**, **b**) Subcutaneous tumour weights also did not differ in mice injected with HepG2-WISP2 and HepG2-Vector. (**B**, **a**, **b**) Higher expression of WISP2 were acting as a protective factor, especially in HCC patients without alcohol intake. (**C**) WISP2 expression was significantly negatively correlated with tumour purity (**a**), and was significantly correlated with the specific marker of vascular endothelial cells (**b**) and fibroblast (**c**) in TIMER database. (**D**) The interactions between WISP2 and ACTA2 were evaluated using GeneMANIA database. (**E**) Increased numbers of fibroblasts and fibro-collagen deposition were positive correlated with the expression of WISP2 in HCC. (**F**) Human liver cancer tissue microarrays confirmed a positive correlation between WISP2 and α-SMA.

In this section, the role of WISP2 was evaluated *in vivo*. While, in nude mouse models, subcutaneous tumour growth after 4 weeks did not differ in mice injected with Hep3B-WISP2 and Hep3B-Vector (1.23 ± 0.15 mm vs. 1.33 ± 0.12 mm, *p* = 0.2354; Figure 3A, 3a). And subcutaneous tumour growth also did not differ in mice injected with HepG2-WISP2 and HepG2-Vector (1.40 ± 0.14 mm vs. 1.58 ± 0.15 mm, *p* = 0.103; Figure 3A, 3b). This finding was in direct contrast to our *in vitro* results. Additionally, we classified the patients into subgroups according to alcohol intake history, the major aetiologies of liver cancer in western countries. In patients without alcohol intake history, patients with high WISP2 expression showed significantly longer OS and RFS compared with those with low WISP2 expression (Figure 3B, 3a). While, there was no significant difference in OS and RFS between high and low WISP2 expression in patients with alcohol intake history (Figure 3B, 3b). It is now clear that alcohol consumption is closely related to liver fibrosis [20]. Thus, we sought to examine tumour microenvironmental influences on the WISP2 effects.

Stromal cells play an important role in cancer progression, and tumour-infiltrating lymphocytes are an independent predictor of survival in cancers, especially in HCC. Therefore, we first investigated whether WISP2 expression was correlated with tumour purity in HCC using TIMER database. Indeed, WISP2 expression was significantly negatively correlated with tumour purity (*r* = –0.517, *p* = 5.01e-25), indicating that more stromal cells were present in HCC tissues with high WISP2 expression. In addition, WISP2 expression was weakly correlated with infiltrating lymphocytes, such as B cells (*r* = 0.068, *p* = 2.08e-1), CD8^+^ T cells (*r* = 0.178, *p* = 9.37e-4), CD4^+^ T cells (*r* = 0.217, *p* = 4.99e-5), macrophages (*r* = 0.181, *p* = 7.82e-4), neutrophils (*r* = 0.113, *p* = 3.61e-2), and dendritic cells (*r* = 0.174, *p* = 1.31e-3; Figure 3C, 3a). We also explored the relationship between WISP2 expression and infiltrating vascular endothelial cells and fibroblasts. Interestingly, WISP2 expression was correlated with the specific marker of vascular endothelial cells marker CD34 (*r* = 0.373, *p* = 1.03e-13), CD31(PECAM1, *r* = 0.479, *p* = 1.25e-22), VCAM1 (*r* = 0.156, *p* = 2.54e-3), NT5E (*r* = –0.065, *p* = 2.15e-1), and ESM1 (*r* = –0.34, *p* = 2.54e-3; Figure 3C, 3b). And the most relevant are WISP2 expression and the fibroblast markers S100A4 (*r* = 0.437, *p* = 1.03e-18) and α-SMA (ACTA2; *r* = 0.544, *p* = 5.3e-30), and Vimentin (Vim; *r* = 0.579, *p* = 1.34e-34; Figure 3C, 3c). We next examined the interactions between WISP2 and other proteins using GeneMANIA database for cancer genomics database, illuminating a strong interaction among WISP2, connective tissue growth factor (CTGF), and ACTA2 (Figure 3D). These results suggest that WISP2 expression reflects fibroblast infiltration in the tumour.

Additionally, we further examined the co-expression of WISP2 and α-SMA in subcutaneous tumour tissues. Increased numbers of fibroblasts and fibro-collagen deposition were positive correlated with the expression of WISP2 in HCC (Figure 3E). Human liver cancer tissue microarrays confirmed a positive correlation between WISP2 and α-SMA (Figure 3F). These results suggested that WISP2 played a negative role in tumour purity by promoting fibroblast infiltration into the tumour microenvironment.

### Upregulation of WISP2 in HCC cells significantly alters gene expression profiles

**Figure 4:**
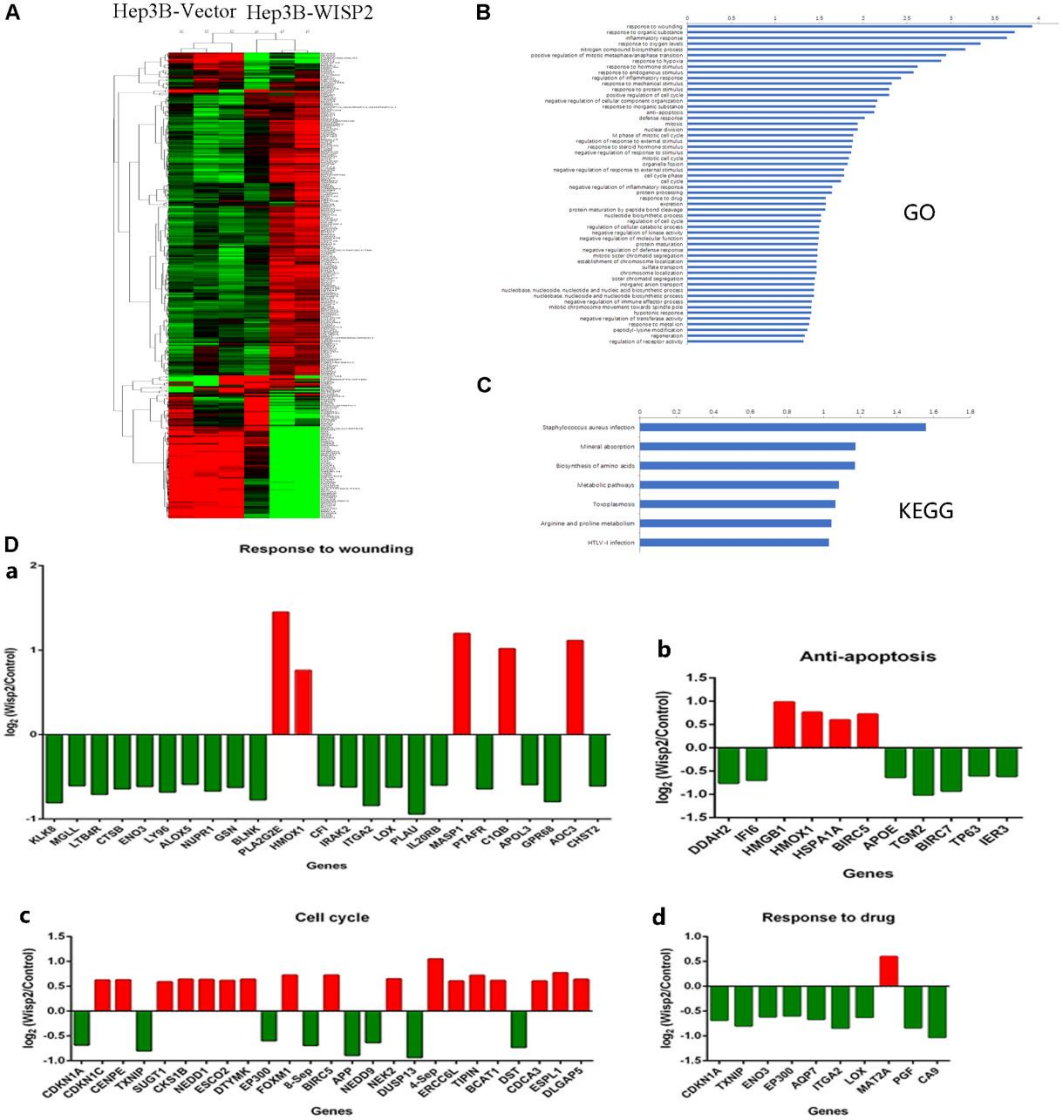
**Gene expression profiles were significantly altered in HCC cells that overexpressed WISP2.** (**A**) Heatmap shows the gene expression profiles of Hep3B cells with and without overexpression of WISP2. (**B**) The differentially expressed genes were evaluated using Gene Ontology analysis. (**C**) The differentially expressed genes were evaluated by KEGG pathway analysis. (**D**) Differentially expressed genes were found to be involved in wounding healing (**a**), anti-apoptosis (**b**), cell cycle (**c**), and drug resistance (**d**).

Gene expression profiles were significantly altered in Hep3B cells that overexpressed WISP2 compared to vector control cells, with 415 differentially expressed genes (Figure 4A). We analysed these altered genes using Gene Ontology (GO, Figure 4B) and Kyoto Encyclopedia of Genes and Genomes (KEGG, Figure 4C) pathway enrichment. According to GO analysis, 25 genes involved in wound healing, were significantly changed, and 80% of these were inhibited by WISP2 overexpression. The downregulated genes included *KLK8*, *MGLL*, *ENO3*, *ALOX5*, *BLNK*, *CFI*, *IRAK2*, *GPR68*, and *APOL3* (Figure 4D, 4a). With respect to anti-apoptosis factors, 11 genes were significantly altered by WISP2 overexpression (Figure 4D, 4b). Twenty five genes related to the cell cycle were significantly changed, and 8 core genes were significantly downregulated (Figure 4D, 4c). Of the 10 drug resistance-related genes that were altered, nine were downregulated by WISP2 overexpression (Figure 4D, 4d). KEGG pathway enrichment analysis revealed significant alterations in seven important pathways, including Staphylococcus aureus infection, Mineral absorption, Biosynthesis of amino acids, Metabolic pathways, Arginine and proline metabolism, Toxoplasmosis, and HTLV-I infection. High-mobility group protein box1 (HMGB1) is a pivotal factor in the development and progression of many types of tumours, which is closely correlated with tumour-mediated inflammation microenvironment [21]. Interestingly, HMGB1 was significantly upregulated after WISP2 overexpression in HCC cells.

### Enhanced fibroblast infiltration is related to upregulated HMGB1 in the presence of WISP2 overexpression in HCC

**Figure 5:**
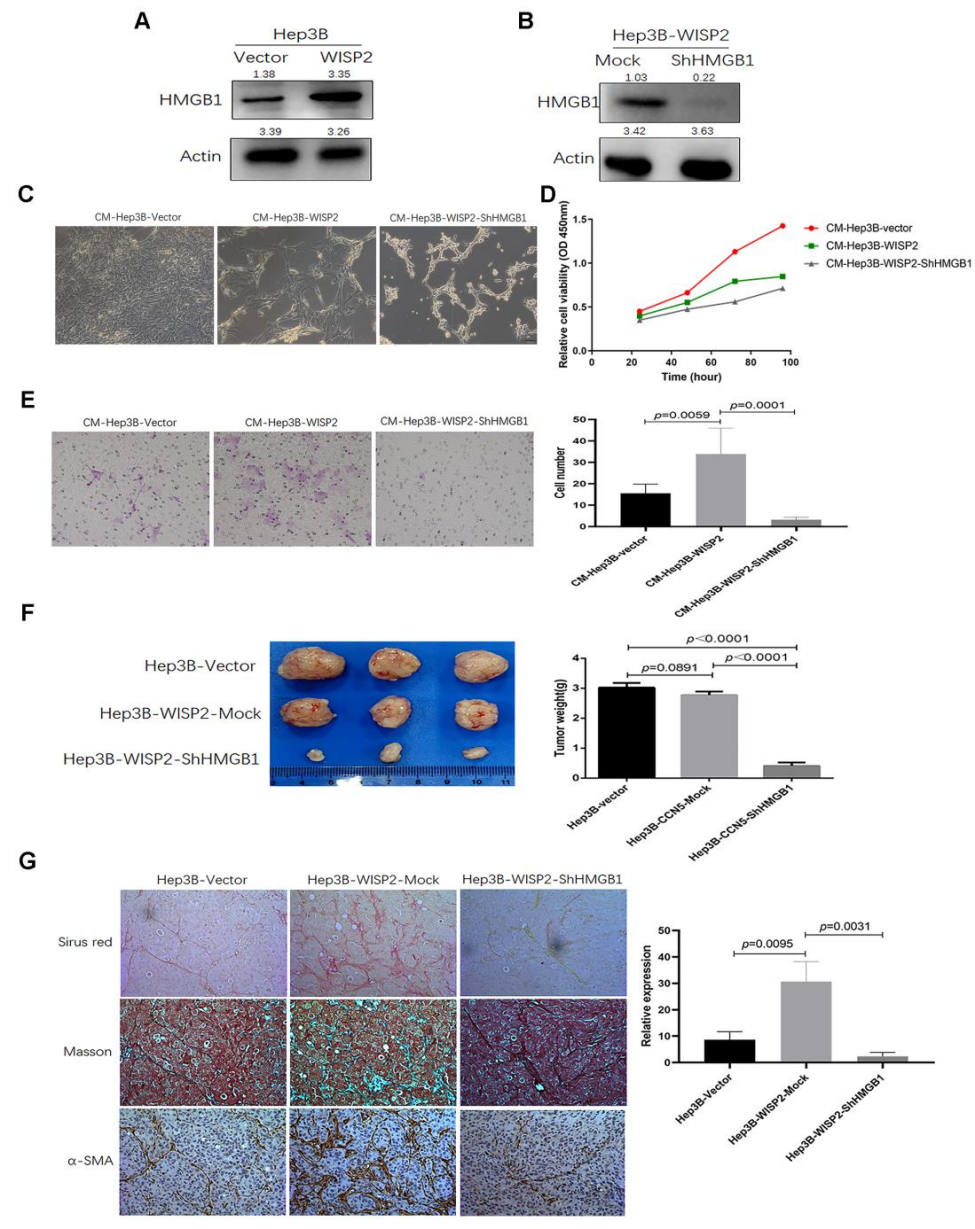
**The infiltration of fibroblast cells into HCC tissues is related to HMGB1 after overexpression of WISP2.** (**A**) HMGB1 was significantly upregulated after overexpression of WISP2 at the protein level using immunoblotting in HCC. (**B**) Immunoblotting was performed to determine the downregulated expression of HMGB1 in Hep3B-WISP2-shHMGB1. (**C**, **D**) Hepatic stellate LX2 cells treated with CM from Hep3B-WISP2 or CM from Hep3B-WISP2-shHMGB1, exhibited inhibited proliferation ability. (**E**) LX2 cells treated with CM from Hep3B-WISP2 exhibited enhanced migration ability, while exhibited inhibited migration ability after treated with CM from Hep3B-WISP2-shHMGB1. (**F**) Subcutaneous tumours in nude mice were induced via inoculation with Hep3B-Vector, Hep3B-WISP2-Mock, and Hep3B-WISP2-ShHMGB1 HCC cells, and the weights of tumours from Hep3B-WISP2-ShHMGB1 cells were significantly decreased. (**G**) Tumours generated from the Hep3B-WISP2-ShHMGB1 cells exhibited significantly decreased α-SMA expression and fibro-collagen deposition.

**Figure 6:**
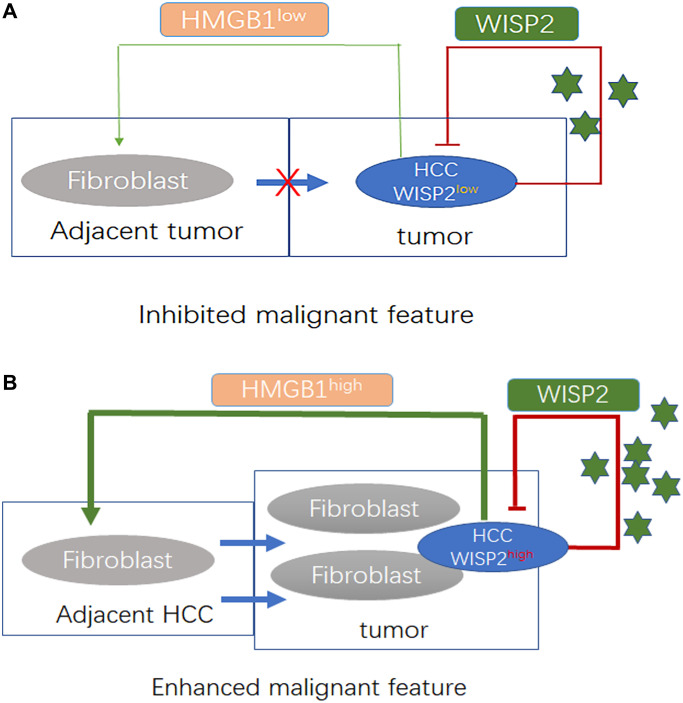
**WISP2 is a tumour suppressor that is influenced by tumour purity and fibroblast infiltration related to the expression of HMGB1 in HCC.** (**A**) Decreased fibroblast infiltration into HCC tissue was related to the downregulation of HMGB1 in HCC in the presence of low expression of WISP2. Under these conditions, HCC exhibited a restrained state. (**B**) Increased number of fibroblasts in HCC tissue was related to the upregulation of HMGB1 induced by WISP2. Under these conditions, HCC exhibited a proliferative state.

As determined in the gene expression profiles following WISP2 overexpression in HCC cells, HMGB1 was significantly upregulated, and this upregulation was found to be strongly correlated with cirrhosis in our previous study [21]. In this study, we first validated these gene array assays at the protein level via immunoblotting and confirmed the upregulation of HMGB1 in the presence of WISP2 overexpression (Figure 5A). Next, we generated a Hep3B-WISP2 cell line with stably downregulated expression of HMGB1 (Hep3B-WISP2-shHMGB1; Figure 5B). Hepatic stellate LX2 cells treated with conditioned medium (CM) from Hep3B-WISP2-shHMGB1 exhibited inhibited proliferation ability (Figure 5C, 5D). While, LX2 cells treated with CM from Hep3B-WISP2-shHMGB1 exhibited enhanced migration ability (3.17 ± 1.17 vs. 33.83 ± 12.16, *p* = 0.0001; Figure 5E). We then produced subcutaneous tumours in nude mice via transplantation of Hep3B-Vector, Hep3B-WISP2-Mock, and Hep3B-WISP2-shHMGB1 cells. After 4 weeks, weights of the subcutaneous tumours from Hep3B-WISP2-Vector and Hep3B-WISP2-Mock cells were also not significantly different (2.78 ± 0.12 g vs. 3.02 ± 0.16 g, *p* = 0.0819), while the weights of tumours from Hep3B-WISP2-ShHMGB1 cells were significantly decreased (0.42 ± 0.11 g vs. 2.78 ± 0.12 g, *p* < 0.0001; Figure 5F). We also found increased expression of α-SMA and fibro-collagen deposition in tumour tissues generated from Hep3B-WISP2-Mock cells, while tumours generated from the Hep3B-WISP2-ShHMGB1 cells exhibited significantly decreased α-SMA expression and fibro-collagen deposition (Figure 5G). Thus, HMGB1 is involved in the increased fibroblast infiltration that results from WISP2 overexpression (Figure 6).

## DISCUSSION

HCC is the fourth leading cause of cancer death worldwide according to global cancer statistics 2018 [22], and alcohol abuse are considered to be pathogenic factors for HCC in western countries [23, 24]. Although early diagnosis and surgical resection are primary anti-tumor strategies, the prognosis of cancer patients remains generally dismal, with a 5-year overall survival rate of only 50–70%, and the unfavorable outcomes attributed to the high frequency of tumor recurrence, metastasis, and therapeutic resistance [25]. Therefore, continued identification of new molecules for early survival prediction and the development of molecular targeted therapy are still urgently needed.

Cellular communication network (CCN) family are scaffolding proteins that may govern as localized multitasking signal integrators in tumours and the associated TME [26]. CCN proteins are involved in many vital biological functions, including angiogenesis, fibrosis, and tissue regeneration and repair [14]. In human cancers, the expression levels of CCN proteins are closely correlated with in regulating tumor cellular function and the TME [27]. As CCN5 lacks a CT domain, this striking difference in structure compared with other CCN family members may allow it to have unique functional roles. Like its family members, however, previous studies reported inconsistent roles of WISP2 in carcinogenesis. In HCC, the role of WISP2 in tumor progression also remains unclear. Previously, no significant difference in WISP2 expression was identified between HCC tumours and matched normal liver samples [13], indicating that the role of WISP2 in HCC tumour progression remains unresolved. In the present study, data from TIMER and LinkedOmics databases revealed that levels of WISP2 were lower in tumour tissues than in normal liver tissues, and high expression of WISP2 was associated with better prognosis in HCC patients, as revealed by the Kaplan–Meier plotter, indicating WISP2 is a protective factor in HCC. In addition, in HCC patients without history of alcohol intake, high expression of WISP2 indicated significantly better prognosis, indicating that the effects of WISP2 may be influenced by the microenvironment. *In vitro* studies also showed that upregulation of WISP2 in HCC is related to inhibition of the malignant phenotype, although such inhibition of proliferation *in vivo* was not obvious.

Because the *in vivo* and *in vitro* studies were inconsistent, we sought to explore potential influences in the tumour microenvironment. Solid tumours are complex entities, as they are surrounded by a heterogeneous array of extracellular matrix and various stromal cells that play important roles in cancer progression. Specifically, tumour-infiltrating lymphocytes are an independent predictor of survival in cancers [28]. In the present study, WISP2 expression was negatively correlated with tumour purity in HCC. And we found WISP2 expression was weakly correlated with infiltrating lymphocytes, including CD8^+^ T cells, CD4^+^ T cells, B cells, neutrophils, dendritic cells, and macrophages, while WISP2 was positively correlated with the number of fibroblasts in the TME. The co-expression of WISP2 and the fibroblast marker α-SMA in subcutaneous tumour tissue and HCC tissue microarrays confirmed this relationship. Therefore, we hypothesized that the inconsistency of results *in vitro* and *in vivo* was due to fibroblast infiltration in the TME.

Accumulating evidence supports the concept that cirrhosis is one of the key factors that promotes HCC [29]. And according to the reports, the role of WISP2 appeared to have paradoxical effects in stromal cells. Grünberg et al. [30] showed that WISP2 activates the canonical WNT pathway and increased β-catenin levels via targeting LRP5/6 phosphorylation in mesenchymal cells. In the myocardium, WISP2 can reverse established cardiac fibrosis via inhibition of enhanced apoptosis of myofibroblasts [31]. In the current study, we demonstrated that CM from an HCC cell line that was engineered to overexpress WISP2 significantly inhibited proliferation of LX2, and after downregulation of HMGB1 in these cells, an additional significant decrease in proliferation was observed. While, it is surprised that we proved the CM from an HCC cell line that was engineered to overexpress WISP2 significantly increase the migration ability of LX2, and the trends was reversed in the HCC cell line with stably downregulated expression of HMGB1.

HMGB1 plays a role in several cellular processes, including inflammation, cell differentiation and migration [32]. In our previous study, the upregulation of HMGB1 was found to be strongly correlated with cirrhosis in HCC [21]. According to our screening results, HMGB1 was one of the significantly upregulated genes after WISP2 overexpression in HCC cells, we take HMGB1 as a follow-up research target. In the follow-up study, the weights of subcutaneous tumours generated from these engineered HCC cells in nude mice were also significantly decreased after downregulation of HMGB1 expression in the presence of WISP2 overexpression, indicating that HMGB1 is one of the key factors involved in reducing the anticancer efficiency of WISP2. Additionally, we observed decreased expression of the fibroblast biomarker α-SMA in tumour tissues with low expression of HMGB1. These results are in line with previous reports that upregulation of HMGB1 is associated with inflammatory pathogenesis, with enhanced local inflammation and fibrosis [33].

## CONCLUSIONS

We propose that as high expression of WISP2 is associated with better prognosis in HCC, WISP2 can serve as a prognostic biomarker and the prediction efficiency is influenced by tumour purity with fibroblast infiltration. And the enhanced infiltration of fibroblasts is related to upregulated expression of HMGB1, that results from WISP2 overexpression and weakens the anticancer effects of WISP2 (Figure 6). The findings shed light on the dual roles of WISP2 in HCC, suggesting that WISP2 up-regulation combined with HMGB1 inhibition may serve as an effective therapeutic strategy for better prognosis in HCC. While our study was only limited to liver cancer, and lack of deeper mechanistic knowledge of the regulatory relationship between HMGB1 and chemokines from HCC. Therefore, several fundamental questions remain to be answered concerning the mechanism of WISP2 regulating HMGB1 and chemokines in the further study.

### Availability of data and material

The datasets used and/or analyzed and materials developed during the current study are available from the the repository of TCGA (https://portal.gdc.cancer.gov) and corresponding author by reasonable request.

## Supplementary Materials

Supplementary Materials and Methods

## References

[1] Chen W, Zheng R, Baade PD, Zhang S, Zeng H, Bray F, Jemal A, Yu XQ, He J. Cancer statistics in China, 2015. CA Cancer J Clin. 2016; 66:115–32. 10.3322/caac.21338 26808342

[2] Llovet JM, De Baere T, Kulik L, Haber PK, Greten TF, Meyer T, Lencioni R. Locoregional therapies in the era of molecular and immune treatments for hepatocellular carcinoma. Nat Rev Gastroenterol Hepatol. 2021; 18:293–313. 10.1038/s41575-020-00395-0 33510460

[3] Jia Q, Xu B, Zhang Y, Ali A, Liao X. CCN Family Proteins in Cancer: Insight Into Their Structures and Coordination Role in Tumor Microenvironment. Front Genet. 2021; 12:649387. 10.3389/fgene.2021.649387 33833779 PMC8021874

[4] Perbal B. CCN proteins are part of a multilayer complex system: a working model. J Cell Commun Signal. 2019; 13:437–39. 10.1007/s12079-019-00543-5 31848849 PMC6946776

[5] Jia Q, Dong Q, Qin L. CCN: core regulatory proteins in the microenvironment that affect the metastasis of hepatocellular carcinoma? Oncotarget. 2016; 7:1203–14. 10.18632/oncotarget.6209 26497214 PMC4811454

[6] Russo JW, Castellot JJ. CCN5: biology and pathophysiology. J Cell Commun Signal. 2010; 4:119–30. 10.1007/s12079-010-0098-7 21063502 PMC2948116

[7] Banerjee SK, Banerjee S. CCN5/WISP-2: A micromanager of breast cancer progression. J Cell Commun Signal. 2012; 6:63–71. 10.1007/s12079-012-0158-2 22487979 PMC3368018

[8] Davies SR, Watkins G, Mansel RE, Jiang WG. Differential expression and prognostic implications of the CCN family members WISP-1, WISP-2, and WISP-3 in human breast cancer. Ann Surg Oncol. 2007; 14:1909–18. 10.1245/s10434-007-9376-x 17406949

[9] Mason HR, Lake AC, Wubben JE, Nowak RA, Castellot JJ Jr. The growth arrest-specific gene CCN5 is deficient in human leiomyomas and inhibits the proliferation and motility of cultured human uterine smooth muscle cells. Mol Hum Reprod. 2004; 10:181–87. 10.1093/molehr/gah028 14981145

[10] Dhar G, Mehta S, Banerjee S, Gardner A, McCarty BM, Mathur SC, Campbell DR, Kambhampati S, Banerjee SK. Loss of WISP-2/CCN5 signaling in human pancreatic cancer: a potential mechanism for epithelial-mesenchymal-transition. Cancer Lett. 2007; 254:63–70. 10.1016/j.canlet.2007.02.012 17383817

[11] Davies SR, Davies ML, Sanders A, Parr C, Torkington J, Jiang WG. Differential expression of the CCN family member WISP-1, WISP-2 and WISP-3 in human colorectal cancer and the prognostic implications. Int J Oncol. 2010; 36:1129–36. 10.3892/ijo_00000595 20372786

[12] Yang Z, Yang Z, Zou Q, Yuan Y, Li J, Li D, Liang L, Zeng G, Chen S. A comparative study of clinicopathological significance, FGFBP1, and WISP-2 expression between squamous cell/adenosquamous carcinomas and adenocarcinoma of the gallbladder. Int J Clin Oncol. 2014; 19:325–35. 10.1007/s10147-013-0550-9 23592278

[13] Zhang H, Li W, Huang P, Lin L, Ye H, Lin D, Koeffler HP, Wang J, Yin D. Expression of CCN family members correlates with the clinical features of hepatocellular carcinoma. Oncol Rep. 2015; 33:1481–92. 10.3892/or.2015.3709 25571929

[14] Li T, Fan J, Wang B, Traugh N, Chen Q, Liu JS, Li B, Liu XS. TIMER: A Web Server for Comprehensive Analysis of Tumor-Infiltrating Immune Cells. Cancer Res. 2017; 77:e108–10. 10.1158/0008-5472.CAN-17-0307 29092952 PMC6042652

[15] Tang Z, Kang B, Li C, Chen T, Zhang Z. GEPIA2: an enhanced web server for large-scale expression profiling and interactive analysis. Nucleic Acids Res. 2019; 47:W556–60. 10.1093/nar/gkz430 31114875 PMC6602440

[16] Barretina J, Caponigro G, Stransky N, Venkatesan K, Margolin AA, Kim S, Wilson CJ, Lehár J, Kryukov GV, Sonkin D, Reddy A, Liu M, Murray L, et al. The Cancer Cell Line Encyclopedia enables predictive modelling of anticancer drug sensitivity. Nature. 2012; 483:603–07. 10.1038/nature11003 22460905 PMC3320027

[17] Nagy Á, Lánczky A, Menyhárt O, Győrffy B. Validation of miRNA prognostic power in hepatocellular carcinoma using expression data of independent datasets. Sci Rep. 2018; 8:9227. 10.1038/s41598-018-27521-y 29907753 PMC6003936

[18] Warde-Farley D, Donaldson SL, Comes O, Zuberi K, Badrawi R, Chao P, Franz M, Grouios C, Kazi F, Lopes CT, Maitland A, Mostafavi S, Montojo J, et al. The GeneMANIA prediction server: biological network integration for gene prioritization and predicting gene function. Nucleic Acids Res. 2010; 38:W214–20. 10.1093/nar/gkq537 20576703 PMC2896186

[19] Vasaikar SV, Straub P, Wang J, Zhang B. LinkedOmics: analyzing multi-omics data within and across 32 cancer types. Nucleic Acids Res. 2018; 46:D956–63. 10.1093/nar/gkx1090 29136207 PMC5753188

[20] Jepsen P, Kraglund F, West J, Villadsen GE, Sørensen HT, Vilstrup H. Risk of hepatocellular carcinoma in Danish outpatients with alcohol-related cirrhosis. J Hepatol. 2020; 73:1030–36. 10.1016/j.jhep.2020.05.043 32512015

[21] Zhang QB, Jia QA, Wang H, Hu CX, Sun D, Jiang RD, Zhang ZL. High-mobility group protein box1 expression correlates with peritumoral macrophage infiltration and unfavorable prognosis in patients with hepatocellular carcinoma and cirrhosis. BMC Cancer. 2016; 16:880. 10.1186/s12885-016-2883-z 27836008 PMC5106788

[22] Bray F, Ferlay J, Soerjomataram I, Siegel RL, Torre LA, Jemal A. Global cancer statistics 2018: GLOBOCAN estimates of incidence and mortality worldwide for 36 cancers in 185 countries. CA Cancer J Clin. 2018; 68:394–24. 10.3322/caac.21492 30207593

[23] Feng RM, Zong YN, Cao SM, Xu RH. Current cancer situation in China: good or bad news from the 2018 Global Cancer Statistics? Cancer Commun (Lond). 2019; 39:22. 10.1186/s40880-019-0368-6 31030667 PMC6487510

[24] Seitz HK, Bataller R, Cortez-Pinto H, Gao B, Gual A, Lackner C, Mathurin P, Mueller S, Szabo G, Tsukamoto H. Alcoholic liver disease. Nat Rev Dis Primers. 2018; 4:16. 10.1038/s41572-018-0014-7 30115921

[25] Winkler J, Abisoye-Ogunniyan A, Metcalf KJ, Werb Z. Concepts of extracellular matrix remodelling in tumour progression and metastasis. Nat Commun. 2020; 11:5120. 10.1038/s41467-020-18794-x 33037194 PMC7547708

[26] Yeger H, Perbal B. CCN family of proteins: critical modulators of the tumor cell microenvironment. J Cell Commun Signal. 2016; 10:229–40. 10.1007/s12079-016-0346-6 27517291 PMC5055503

[27] Perbal B. The concept of the CCN protein family revisited: a centralized coordination network. J Cell Commun Signal. 2018; 12:3–12. 10.1007/s12079-018-0455-5 29470822 PMC5842208

[28] Quail DF, Joyce JA. Microenvironmental regulation of tumor progression and metastasis. Nat Med. 2013; 19:1423–37. 10.1038/nm.3394 24202395 PMC3954707

[29] Nault JC, Ningarhari M, Rebouissou S, Zucman-Rossi J. The role of telomeres and telomerase in cirrhosis and liver cancer. Nat Rev Gastroenterol Hepatol. 2019; 16:544–58. 10.1038/s41575-019-0165-3 31253940

[30] Grünberg JR, Hammarstedt A, Hedjazifar S, Smith U. The Novel Secreted Adipokine WNT1-inducible Signaling Pathway Protein 2 (WISP2) Is a Mesenchymal Cell Activator of Canonical WNT. J Biol Chem. 2014; 289:6899–907. 10.1074/jbc.M113.511964 24451367 PMC3945351

[31] Jeong D, Lee MA, Li Y, Yang DK, Kho C, Oh JG, Hong G, Lee A, Song MH, LaRocca TJ, Chen J, Liang L, Mitsuyama S, et al. Matricellular Protein CCN5 Reverses Established Cardiac Fibrosis. J Am Coll Cardiol. 2016; 67:1556–68. 10.1016/j.jacc.2016.01.030 27150688 PMC5887128

[32] Siddiqui SS, Dhar C, Sundaramurthy V, Sasmal A, Yu H, Bandala-Sanchez E, Li M, Zhang X, Chen X, Harrison LC, Xu D, Varki A. Sialoglycan recognition is a common connection linking acidosis, zinc, and HMGB1 in sepsis. Proc Natl Acad Sci U S A. 2021; 118:e2018090118. 10.1073/pnas.2018090118 33658363 PMC7958265

[33] Zhao J, Yu J, Xu Y, Chen L, Zhou F, Zhai Q, Wu J, Shu B, Qi S. Epidermal HMGB1 Activates Dermal Fibroblasts and Causes Hypertrophic Scar Formation in Reduced Hydration. J Invest Dermatol. 2018; 138:2322–32. 10.1016/j.jid.2018.04.036 29787749

