# Supplementary material for: Dual roles of WISP2 in the progression of hepatocellular carcinoma: implications of the fibroblast infiltration into the tumor microenvironment
Source: Aging (Albany NY). 2021 Sep 8;13(17):21216–31. doi: 10.18632/aging.203424 (PMC8457598; doi:10.18632/aging.203424)
Supplement: Supplementary Materials and Methods [file aging-13-203424-s001.pdf]

SUPPLEMENTARY MATERIALS AND METHODS

Vector construction, transfection, and lentivirus transduction

Human full-length WISP2 cDNA was obtained from GeneCards (Shanghai, China) and cloned into the pCDH lentiviral expression vector (System Biosciences, Palo Alto, CA, USA). The amplified fragment was inserted into the pCDH plasmid (between *XbaI* and *EcoRI* sites) using the In-Fusion HD Cloning Kit (Takara, Tokyo, Japan). High-mobility group protein 1 (HMGB1) shRNA and scramble plasmids were obtained as a gift from the Institutes of Biomedical Sciences at Fudan University (Shanghai, China), and each was in the lentiviral expression plasmid PLKO.1. Stable cell lines were created by antibiotic selection with puromycin for a week, beginning 72 h after transduction.

Animal models

A total of 21 male BALB/c nu/nu mice (aged 4–6 weeks and weighing approximately 20 g) and raised in a controlled environment with 25°C under standard pathogen-free conditions and a natural light/dark cycle (morning 8:00; afternoon 8:00), and were provided with water and standard diet. Hep3B-Vector, Hep3B-WISP2, HepG2-Vector, HepG2-WISP, and Hep3B-WISP2-Mock, and Hep3B-WISP2-shHMGB1 cells were implanted subcutaneously into the upper left flank region of mice to establish subcutaneous xenografts. Tumour weights were evaluated 4 weeks after the treatments. The intraperitoneal injection of pentobarbital (5 mg/kg) combined with cervical spondylolisthesis was used for euthanasia of the mice after the study. The study protocol was approved by the Medical Experimental Animal Care Commission of Northwest Polytechnical University, and all methods were performed in accordance with the relevant guidelines.

Patients and tissue microarray analysis

Immunohistochemical analysis of tissue microarrays included 186 samples obtained from HCC patients. Written consent was obtained from patients who received curative resection at the Liver Cancer Institute of Zhongshan Hospital of Fudan University between January 2004 and December 2006. Post-surgical follow-up occurred until December 2013, with a median follow-up period of 63 months (range, 0–110 months). Curative resection was defined as complete resection of tumour nodules with clean post-surgical margins. Histopathological diagnosis was performed according to WHO criteria. Ethical approval was obtained from the Research Ethics Committee of Fudan University.

Antibodies, cell lines and preparation of conditioned media (CM)

Monoclonal antibodies were used for immunoblot and/or Immunohistochemistry analysis, including WISP2, ki67, α-SMA, E-cadherin, and Vimentin (Abcam, Cambridge, MA, USA), HMGB1, Snail, and β-Actin (Proteintech, Chicago, IL, USA).

The HCC cell line Hep3B and hepatic stellate cell line LX2 were obtained from the Liver Cancer Institute of Fudan University (Shanghai, China). All cell lines were cultured in Dulbecco's Modified Eagle's Media (DMEM; GIBCO, Grand Island, NY, USA) containing 10% foetal bovine serum (FBS; GIBCO) at 37°C in a humidified incubator with 5% CO_2.

The Hep3B-WISP2, Hep3B-Vector, and Hep3B-WISP2-shHMGB1 cell lines were plated in T_{75} flasks (1×10^6 cells). Medium was replaced with 6 mL of fresh DMEM containing 2% FBS the next day. After 24 h, CM were centrifuged at 1,000 × g, and the supernatants were collected and designated CM-Hep3B-WISP2, CM-Hep3B-Vector, and CM-Hep3B-WISP2-shHMGB1. Finally, CM from these cell lines were used for treating LX2 cells.

Migration and invasion assays

Migration of Hep3B-WISP2 and Hep3B-Vector were evaluated in Boyden chambers containing membranes with 8.0-μm pores in 24-well plates (Corning, Tewksbury, MA, USA). Cells were seeded into the upper chamber of each well in serum-free DMEM (6×10^4 cells/well). DMEM containing 2% FBS was added to the lower chamber of each well. After 24 h, cells on the underside of the membrane were stained with Giemsa (Sigma Chemical Company, St. Louis, MO, USA), counted, and photographed at 200× magnification. Cell invasion assays were performed similarly, except that 80 μL Matrigel (BD Biosciences, San Jose, CA, USA) was added to each well 6 h before cells were seeded onto the membrane.

Proliferation assessment

To investigate the proliferative effect of WISP2 on HCC cells, Hep3B-WISP2 and Hep3B-Vector (1×10^3

cells/well) were plated in 6-well plates and cultured with DMEM containing 10% FBS. Culture medium was replaced every 3 d, and the colonies were fixed with ice-cold 4% paraformaldehyde after 14 d. Cells were stained with Giemsa (Sigma, St. Louis, MO, USA) and photographed at ×5 magnification.

Hep3B-WISP2 and Hep3B-Vector were plated in 96-well plates (3×10^3 cells/well) and cultured for 24, 48, 72, and 96 h. Cell proliferation assays were carried out with the Cell Counting Kit 8 (CCK8; Dojindo). Results were expressed as the absorbance of each well at 450 nm (OD450).
